# Multistate Outbreak of *Salmonella* Anatum Infections Linked to Imported Hot Peppers — United States, May–July 2016

**DOI:** 10.15585/mmwr.mm6625a2

**Published:** 2017-06-30

**Authors:** Rashida Hassan, Joshua Rounds, Alida Sorenson, Greg Leos, Jeniffer Concepción-Acevedo, Taylor Griswold, Adiam Tesfai, Tyann Blessington, Cerise Hardy, Colin Basler

**Affiliations:** ^1^ National Center for Emerging and Zoonotic Infectious Diseases, Division of Foodborne, Waterborne, and Environmental Diseases, CDC; ^2^Minnesota Department of Health; ^3^Minnesota Department of Agriculture; ^4^Texas Department of State Health Services; ^5^Food and Drug Administration, Silver Spring, Maryland.

Foodborne salmonellosis causes an estimated 1 million illnesses and 400 deaths annually in the United States (*1*). *Salmonella* Anatum is one of the top 20 *Salmonella* serotypes in the United States. During 2013–2015 there were approximately 300–350 annual illnesses reported to PulseNet, the national molecular subtyping network for foodborne disease surveillance. In June 2016, PulseNet identified a cluster of 16 *Salmonella* Anatum infections with an indistinguishable pulsed-field gel electrophoresis (PFGE) pattern from four states.* In April 2016, the same PFGE pattern had been uploaded to PulseNet from an isolate obtained from an Anaheim pepper, a mild to medium hot pepper. Hot peppers include many pepper varieties, such as Anaheim, jalapeño, poblano, and serrano, which can vary in heat level from mild to very hot depending on the variety and preparation. This rare PFGE pattern had been seen only 24 times previously in the PulseNet database, compared with common PFGE patterns for this serotype which have been seen in the database hundreds of times. Local and state health departments, CDC, and the Food and Drug Administration (FDA) investigated to determine the cause of the outbreak. Thirty-two patients in nine states were identified with illness onsets from May 6–July 9, 2016. Whole-genome sequencing (WGS) was performed to characterize clinical isolates and the Anaheim pepper isolate further. The combined evidence indicated that fresh hot peppers were the likely source of infection; however, a single pepper type or source farm was not identified. This outbreak highlights challenges in reconciling epidemiologic and WGS data, and the difficulties of identifying ingredient-level exposures through epidemiologic investigations alone.

## Epidemiologic Investigation

During June, local and state health departments in seven states interviewed patients with standard foodborne illness questionnaires. By June 29, 14 patients had been interviewed; commonly reported foods eaten in the week preceding illness included tomatoes (71% of respondents); pork (64%); avocado/guacamole (57%); jalapeños, a hot pepper that can vary from mild to hot heat (36%); and cantaloupe (36%). These exposures were compared with the 2006–2007 FoodNet Population Survey, which summarizes data on foods eaten by a sample of healthy persons.^†^ The only food exposure reported significantly more frequently than expected among patients was avocado/guacamole (p = 0.01); however, because the FoodNet Population Survey does not include questions on jalapeños, it was not possible to make a comparison for that exposure. Seven of the 14 interviewed patients reported eating at Mexican-style restaurants in the week preceding illness onset.

The lack of a strong hypothesis for the outbreak source led CDC to propose open-ended interviews by a single interviewer. Open-ended interviews are unstructured, conversational interviews that sometimes identify uncommon exposures because they gather more detailed information than that typically obtained from standard interviews.^§^ CDC completed open-ended interviews with nine patients, including seven from Texas, one from Colorado, and one from Illinois. Concurrently, Minnesota investigators conducted open-ended interviews with eight patients in Minnesota and shared exposure information with CDC.

**FIGURE 1:**
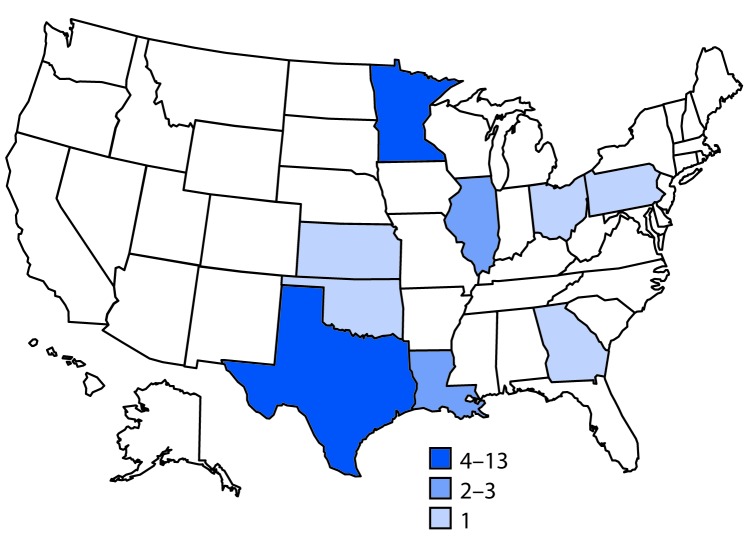
Number of persons (N = 32) infected with the outbreak strain of *Salmonella* Anatum, by state — United States, May 6–July 9, 2016

**FIGURE 2:**
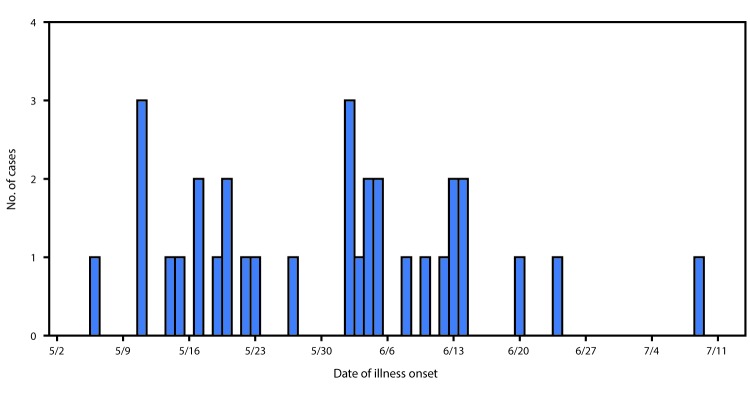
Number of persons (N = 32) infected with the outbreak strain of *Salmonella* Anatum, by date of illness onset — nine states,* May 6–July 9, 2016 *Georgia, Illinois, Kansas, Louisiana, Minnesota, Ohio, Oklahoma, Pennsylvania, and Texas.

A case of *Salmonella* Anatum gastroenteritis was defined as infection with an outbreak strain of *Salmonella* Anatum in a person with onset of diarrheal illness during May 6–July 9, 2016. In total, 32 cases from nine states were identified^¶^ (Figure 1). The median patient age was 36 years (range = 4–79 years); 19 (59%) were female. Illness onset dates ranged from May 6 to July 9 (Figure 2). Among 25 patients for whom information on hospitalization was available, eight (32%) were hospitalized; no deaths were reported. Among 18 patients for whom information from initial or open-ended interviews was available, 14 reported eating, or possibly eating fresh hot peppers, or reported eating an item containing fresh hot peppers. Nine patients reported eating peppers at restaurants, two reported eating peppers both at restaurants and at home, and three did not specify a location. Among the 14 patients who had eaten peppers, 11 reported eating, or possibly eating jalapeños. No patient reported eating Anaheim peppers; most had never heard of an Anaheim pepper.

One illness subcluster was identified consisting of two patients who did not know one another and ate at the same Mexican-style restaurant in Texas in the week preceding illness. Both dined there on the same day and consumed multiple common food items, including steak, eggs, rice, beans, mild salsa, and pico de gallo (a fresh salsa made with chopped tomatoes), hot peppers, and other fresh ingredients. The only fresh hot peppers included in reported meal items were jalapeños, used in both the pico de gallo and mild salsa. The restaurant used serrano and poblano peppers in other dishes, but neither patient reported eating these items. Because the epidemiologic evidence supported hot peppers in general, but not Anaheim peppers specifically, investigators explored multiple hot pepper types as possible outbreak vehicles.

## Traceback Investigation

**FIGURE 3:**
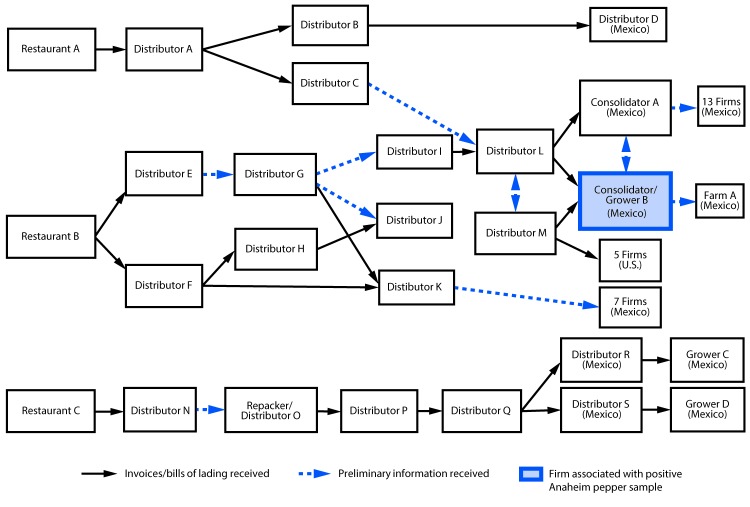
*Salmonella* Anatum outbreak informational traceback flow diagram for fresh hot peppers — United States, May–July 2016

Local and state investigators visited restaurants where patients reported consuming peppers. They collected recipes for reported menu items, including salsa, and reviewed invoices to identify common ingredients. To identify the source of hot peppers, FDA conducted traceback (the process of tracing a food from point-of-service to its origin or manufacturer source) from three restaurants in Minnesota and Texas where patients reported eating. Two of the three restaurants received peppers from a consolidator/grower in Mexico (consolidator/grower B) (Figure 3), which exported Anaheim peppers to the United States in April 2016. Consolidators pool foods from different growers or growing locations; this designation is also used if some growers/growing locations are unknown.** The third restaurant received peppers from various firms in Mexico; however, this restaurant had received peppers from consolidator/grower B before this outbreak. Because of the complicated supply chain for peppers and the extensive mixing of peppers from different suppliers, repacking, and reselling of product, FDA was unable to identify a single source farm or point of contamination for peppers.

In April 2016, before the identification of cases and as part of routine surveillance cultures of produce, the FDA isolated this strain of *Salmonella* Anatum from an Anaheim pepper sample. This Anaheim pepper was collected from consolidator/grower B, which supplied two restaurants reported to have been visited by patients in this outbreak. FDA collected seven additional samples of hot peppers, including serrano, habanero, jalapeño, and bell peppers, from consolidator/grower B as part of the outbreak investigation; none yielded *Salmonella*.

## Laboratory Investigation

Representative clinical isolates and the Anaheim pepper isolate were further characterized by WGS.^††^ High-quality single-nucleotide polymorphism (hqSNP) analysis indicated that 19 clinical isolates and the Anaheim pepper isolate differed by 0–3 hqSNPs, suggesting they were highly related genetically. This strong laboratory evidence was key to aiding in interpretation of the epidemiologic data.

## Public Health Response

On June 21, 2016, before the epidemiologic investigation began, FDA placed consolidator/grower B on import alert for Anaheim peppers because they could be contaminated with *Salmonella.* (A product under an import alert is held at the port of entry before being allowed to enter the country. The importer must provide FDA evidence that the product is free from *Salmonella* within 10 business days of detention of the product; otherwise, the product cannot be imported.) There were only two outbreak-associated illnesses reported after the import alert was issued.

## Discussion

In this outbreak, the only food sample yielding *Salmonella* matching the outbreak strain was an Anaheim pepper, which patients did not report consuming, perhaps because they were unfamiliar with this pepper variety or could not identify the pepper variety they consumed at restaurants in salsas or other dishes with chopped peppers. Although only two of the three restaurants included in the informational traceback investigation received peppers from the same consolidator/grower, it is possible that contaminated peppers cross-contaminated other foods or materials along the supply chain, providing a mechanism for the outbreak strain to reach the third restaurant. In addition, it is possible that the third restaurant did receive contaminated peppers during the outbreak timeframe, but the traceback was unable to uncover this because the companies involved kept incomplete records. However, no observations were made to support or refute either of these hypotheses. The strong genetic relationship between the clinical and food isolates, in combination with the epidemiologic and traceback evidence, indicated that fresh hot peppers were the likely source of the outbreak. Nevertheless, it was not possible to implicate one pepper type or source farm.

The epidemiologic investigation relied on review of restaurant-specific recipes, because pepper varieties were difficult to identify when used as ingredients in foods, particularly when prepared at restaurants. In addition, because many common ingredients are consumed in Mexican-style meals, it was difficult to narrow a hypothesis based on epidemiologic information alone. Similar challenges were documented in previous investigations, including a 2008 *Salmonella* Saintpaul outbreak that sickened 1,500 people; investigators ultimately determined that both fresh jalapeño and serrano peppers were outbreak sources after initial evidence indicated tomatoes might have been the source (*2*).

This outbreak highlights the importance of preventing produce contamination to reduce the risk for foodborne illness, especially for foods that are often consumed raw. Many patients consumed peppers at restaurants, often in dishes like fresh salsa, which are served raw. A 2009 study of *Salmonella* in fresh salsa found that chopped jalapeños were more supportive of *Salmonella* growth than some other raw vegetable ingredients when stored at 53°F–69°F (12°C–21°C) (*3*). *Salmonella* survival and growth varied with salsa formulation and were inhibited only in recipes containing both fresh garlic and lime juice (*3*); further research is needed to assess the microbiologic effects of these formulations. For all recipes, no growth was detected at 39°F (4°C ), underscoring the importance of proper temperature controls at restaurants (*3*). All fresh produce, including hot peppers, should be thoroughly washed before preparation and consumption, and refrigerated as soon as possible to prevent the proliferation of bacteria such as *Salmonella*.

This outbreak also highlights new challenges in outbreak investigations when trying to reconcile epidemiologic data with WGS results indicating that clinical and food isolates are genetically closely related to one another. Although WGS can provide additional resolution of the relatedness of isolates, it should not be used as the sole source of evidence (*4*). Careful review of all available epidemiologic, traceback, and laboratory data is critical to determining the source of foodborne outbreaks as enhanced molecular techniques are implemented.

**Summary**

**What is already known about this topic?**

Salmonellosis is the most common bacterial cause of foodborne illness in the United States. *Salmonella* Anatum is one of the top 20 *Salmonella* serotypes in the United States, with approximately 300–350 illnesses reported to PulseNet annually during 2013–2015. Fresh hot peppers have previously been linked to foodborne outbreaks, including a large 2008 *Salmonella* Saintpaul outbreak that sickened 1,500 persons.

**What is added by this report?**

In June 2016, a nine-state outbreak of *Salmonella* Anatum infections was detected, involving 32 patients, with onset of diarrheal illness during May 6–July 9, 2016. The outbreak strain was isolated from an imported Anaheim pepper. The combined epidemiologic, laboratory, and traceback evidence indicated that fresh hot peppers were the likely source of infection, but a single pepper type or source farm could not be identified.

**What are the implications for public health practice?**

This investigation highlights the importance of using epidemiologic and traceback data in concert with whole-genome sequencing results during the course of foodborne outbreak investigations, as well as the utility of open-ended interviews and restaurant-specific recipe review in identifying ingredient-level exposures. Fresh hot peppers are a potential vehicle for *Salmonella* infections; both the complexity of the hot pepper supply chain, as well as the difficulties of identifying specific pepper types through epidemiologic investigations create challenges to investigating outbreaks linked to fresh hot peppers.

## Footnotes

* https://www.cdc.gov/pulsenet.

† https://www.cdc.gov/foodnet/surveys/population.html.

§ https://www.cdc.gov/foodsafety/outbreaks/investigating-outbreaks/investigations/sources.html.

¶ Georgia (one case), Illinois (two), Kansas (one), Louisiana (one), Minnesota (10), Ohio (one), Oklahoma (one), Pennsylvania (one), Texas (13)

** https://www.fda.gov/ForIndustry/ImportProgram/default.htm.

†† https://www.ncbi.nlm.nih.gov/bioproject/369523.
