# *Erratum*: Vol. 66, No. 25

**DOI:** 10.15585/mmwr.mm6631a7

**Published:** 2017-08-11

In the report “Multistate Outbreak of *Salmonella* Anatum Infections Linked to Imported Hot Peppers — United States, May–July 2016,” on page 663, the footnote (^¶ ^**)** at the bottom of the page should have read ^“^Louisiana (**two**)”.

